# Annual dynamics of global remote industrial heat sources dataset from 2012 to 2021

**DOI:** 10.1038/s41597-024-03461-3

**Published:** 2024-06-14

**Authors:** Caihong Ma, Tianzhu Li, Xin Sui, Ruilin Liao, Yanmei Xie, Pengyu Zhang, Mingquan Wu, Dacheng Wang

**Affiliations:** 1 Aerospace Information Research Institute, Chinese Academy of Sciences, Beijing, 100094 China; 2 College of Information, Beijing Forestry University, Beijing, 100094 China; 3 College of Environment and Spatial Informatics, China University of Mining and Technology, Xuzhou, 100094 China; 4 University of Chinese Academy of Sciences, Beijing, 100000 China

**Keywords:** Sustainability, Energy and behaviour

## Abstract

The spatiotemporal distribution of industrial heat sources (IHS) is an important indicator for assessing levels of energy consumption and air pollution. Continuous, comprehensive, dynamic monitoring and publicly available datasets of global IHS (GIHS) are lacking and urgently needed. In this study, we built the first long-term (2012–2021) GIHS dataset based on the density-based spatiotemporal clustering method using multi-sources remote sensing data. A total of 25,544 IHS objects with 19 characteristics are identified and validated individually using high-resolution remote sensing images and point of interest (POI) data. The results show that the user’s accuracy of the GIHS dataset ranges from 90.95% to 93.46%, surpassing other global IHS products in terms of accuracy, omission rates, and granularity. This long-term GIHS dataset serves as a valuable resource for understanding global environmental changes and making informed policy decisions. Its availability contributes to filling the gap in GIHS data and enhances our knowledge of global-scale industrial heat sources.

## Background & Summary

Industry heat sources (IHS) include industrial firms such as cement plants, oil refineries and exploration fields, and chemical processing plants that demand a large amount of primary or secondary energy during manufacturing and release massive heat radiation and greenhouse gases into the air^1–3^. According to an assessment report released by the Intergovernmental Panel on Climate Change (IPCC), carbon emissions are sourced from the energy-intensive industrial sector (EIIS), accounting for approximately 60.4% of the total CO2 emissions in 2019. The value was over 83% in China^4,5^. Understanding the complex interplay between human activities and global changes, including air pollution and the intensification of global warming^6,7^, requires recognizing the significance of IHS as a crucial source of knowledge. Changes in IHS contribute to understanding global environmental changes and regional economic development, ultimately impacting climate change. Therefore, having lengthy time series data of IHS information is critical to understanding global environmental change and contributing to policy decision-making^8,9^. Obtaining more frequent and comprehensive IHS data at the global scale would be highly valuable.

Many scholars and nonprofit organizations or institutions have focused their attention on the global or regional distribution of one or more energy sources/industries. For example, BP company (https://www.bp.com/) and the International Energy Agency (IEA)^10^ (https://www.iea.org/#statistics-data) regularly update energy (coal, oil, gas, electricity, etc.) prospects every year. Tong *et al*.^3^ developed the Global Power Emissions Database (GPED http://meicmodel.org.cn/?page_id=93) from individual power-generating units in 2010. The Emissions and Generation Resource Integrated Database (eGRID)^11^, the China Coal-Fired Power Plant Emissions Database (CPED)^12^ and the India Coal-Fired Power Plant Database (ICPD)^13^ are also available for regional areas. These data typically cover various regions, enabling global or regional research and adhering to stringent data collection and processing standards^14^. However, traditional bottom-up industrial data from publicly available statistics require considerable effort and large costs involved in the administration of surveys^1,15^. Nevertheless, the degree of uncertainty in these data collection efforts varies among industries and geographical areas^16^. These methods are unsuitable for near real-time monitoring at a large scale, particularly for global IHS^17,18^.

Heat sources, such as the flaring of petroleum gas in oil fields and the combustion of fossil fuels in plants (e.g., cement plants, steelworks, etc.) are usually an essential part of production for most heavy industries^1,8^. The thermal anomalies from industrial heat sources can be tracked using satellite remote sensing technologies with large-scale, high-efficiency and low-cost characteristics^19,20^. Presently, data on thermal anomalies from satellite sensors have been widely used in the detection of global-scale self-ignition fire point data. Some examples of these satellites include the Advanced Very High-Resolution Radiometer (NOAA/AVHRR)^21,22^, Along Track Scanning Radiometer (ATSR)^23^, FY3-VIRR^24^, Landsat 8^25^ and Sentinel-2^26^. Thermal anomaly data is then introduced to detect volcanic activity, oil/gas exploitation and IHS. The Suomi National Polar-orbiting Partnership (NPP) Visible Infrared Imaging Radiometer (VIIRS) is a mature nighttime thermal anomaly product that was successfully applied to volcanic activity^27^ and oil and gas exploitation^28^. Liu *et al*.^8^ used NPP VIIRS Nightfire product data (750 m) to identify industrial heat sources by combining time-space-temperature information. As a result, 15,199 global inventories of industrial heat sources (accuracy 77%) operating between 2012 and 2016 in Liu’s product were detected. The NPP VIIRS active fire product (ACF) with a 375-m resolution for daily/nighttime thermal anomalies^22^ was applied to identify IHS based on a density-based spatial-temporal clustering model to detect much smaller IHS in China^1,18^. A total of 4,143 IHS operating between 2012 and 2017 were identified by Ma^1^, and 2,055 high-confidence IHS were identified by Li^18^ in 2016 in China. Flint night light data was introduced to remove biomass heat source objects caused by slash and burn agriculture^29^ in India^30^. Consequently, 711 IHS were found in India between 2012 and 2018. Zhang *et al*.^15^ established 12,114 industrial objects of heat-releasing industries using thermal anomalies produced by a novel and more accurate method employing daily nighttime VIIRS thermal infrared images in 2018 in China. However, all these datasets except that of Liu^8^ are not yet accessible to the general public. Considering the lack of available reference data to validate IHS, two validation strategies (validation of the segmentation robustness and classification accuracy of IHS) were adopted to check part of Liu’s inventory. Therefore, having access to a more frequently updated dataset of IHS at the global scale for long periods, validated individually and made publicly available is highly valuable.

To address the lack of global industrial heat sources (GIHS) data, we developed a novel GIHS product by leveraging long-term ACF and nighttime light (NTL) data along with a density-based spatial-temporal clustering model. Firstly, we acquired and pre-processed ACF and NTL data spanning the period from 2012 to 2021. Subsequently, we employed an adaptive K-means algorithm in a density-based spatial-temporal clustering model to create the initial GIHS dataset. The final GIHS delineation between 2012 and 2021 was accomplished using the max-nighttime light data. To validate the accuracy of our GIHS product, we conducted a comprehensive assessment by various data sources, which involved online high-resolution image screenshots, point of interest (POI) information, and open street maps. Each validation step was carefully examined by a human. In the following sections, we provide a detailed description of the methods utilized and present the results obtained using our newly developed GIHS dataset (10.5281/zenodo.8308133) for the period from 2012 to 2021.

## Methods

**Fig. 1:**
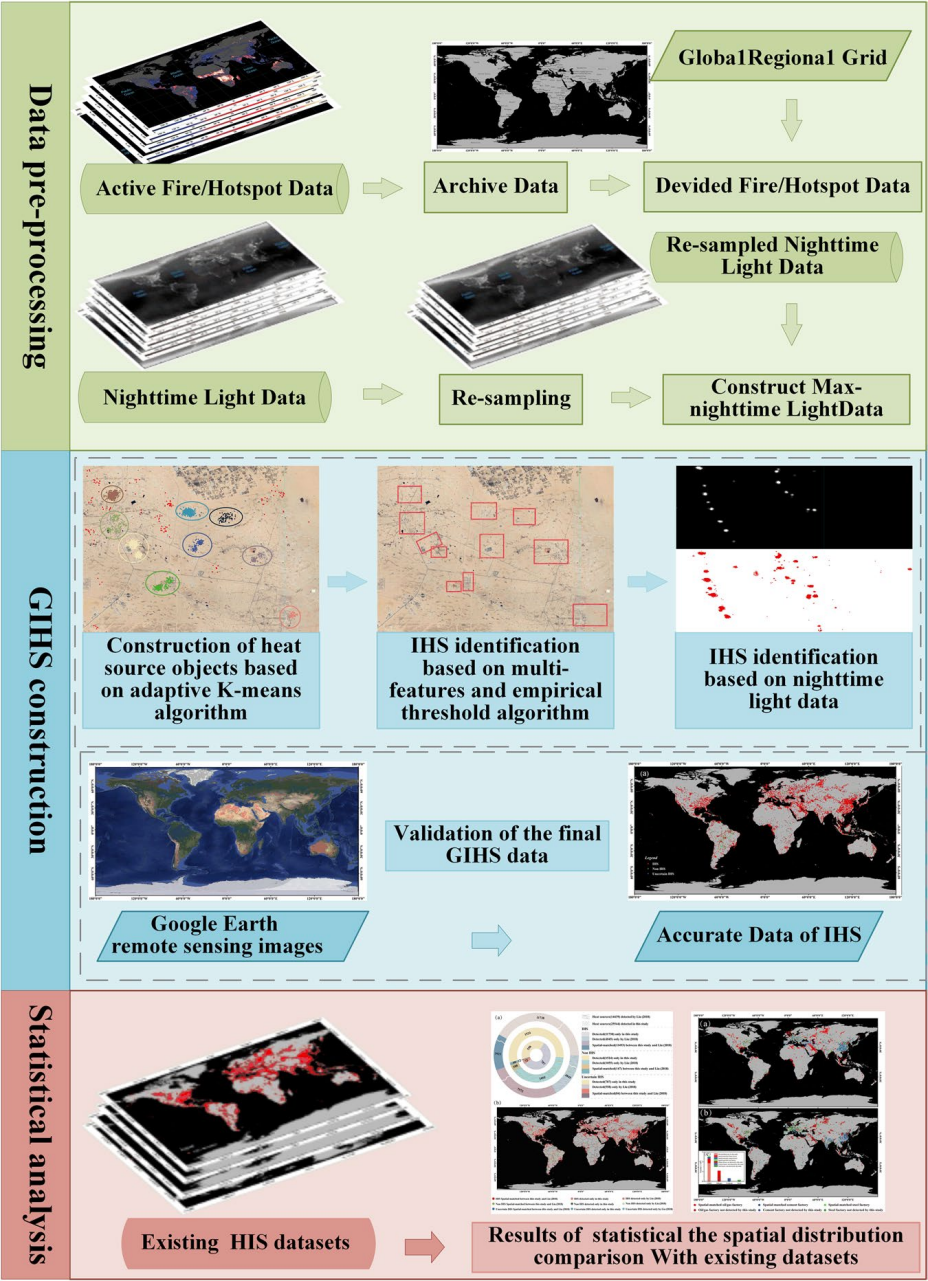
Conceptual representation of the steps involved in the annual dynamics of GIHS from 2012 to 2021 using NPP-VIIRS active fire/hotspot and nighttime light data. More details of the steps can be found in the text.

The main method to construct the annual dynamics of GIHS from 2012 to 2021 using NPP-VIIRS active fire/hotspot and nighttime light data contains three parts: data pre-processing, GIHS construction and Statistical analysis as seen in Fig. 1. More details about those three parts were described as follows.

### Data pre-processing

Considering data using in this paper, this part contained NPP-VIIRS Active Fire/hotspot Data and NPP-VIIRS Nighttime Light Data two pre-processings.

#### NPP-VIIRS Active Fire/hotspot Data Preprocessing

The NPP-VIIRS Active Fire/Hotspot Data (ACF, https://firms.modaps.eosdis.nasa.gov/download/create.php) with 375 m resolution derived from the VIIRS onboard NPP satellite were obtained. It was proposed following the successful application of 375-m resolution data for active fire detection. The first active fires were detected using the VIIRS sensor on 19 January 2012, after the instrument was fully commissioned. From 19 January 2012 to 31 December 2021 (3633 days), 19,661,405 global fire hotspots in 2018 can be seen in Fig. 1a. The ACF product includes a sum of 197,714,128 thermal anomalies.

The computational complexity of the heat source object detection model was positively correlated with the size of the dataset. When the number of fire hotspots increases, the computational complexity will increase exponentially. To reduce the complexity of NPP-VIIRS active fire/hotspot data processing, the data were divided according to the global latitude and longitude 1°x 1°grid. Considering the imbalanced distribution of ACF data, some 1 × 1°grids were divided into four sub-grids when the volume of data was larger than 100,000. In this study, 197,714,128 fire hotspots were divided into 122,460 subhotspots using the global latitude and longitude grid.

#### NPP-VIIRS Nighttime Light Data Preprocessing

For most industrial production activities, the use of lighting is common and necessary. Therefore, superimposed luminous data can be used to verify and filter false heavy industrial heat source areas caused by slash and burning in South America, Asia, and Africa. The spatial resolution of the annual VNL V2 nighttime light data^31^ https://eogdata.mines.edu/nighttime_light/annual/v21/) is 15 arc-seconds, approximately 750 m. The spatial resolution of NPP-VIIRS active fire/hotspot data is 375 m. The yearly VNL V2 nighttime light data were resampled to 375 m to maintain a high spatial resolution.

Most large heavy enterprises were operating between 2012 and 2021. However, there are still many small and medium-sized enterprises and even some large enterprises that were shut down due to economic downturns or policies, such as regional layout and environmental protection, from 2012 to 2021. Taking this into consideration, max-nighttime light data were formed by selecting the maximum value between 2012 and 2021 yearly VNL V2 nighttime lights.

### GIHS construction

We constructed GIHS database based onimproved adaptive K-means algorithm using long-term NPP-VIIRS active fire/hotspot and nighttime light data. Our process for detecting GIHS involved (1) Construction of heat source objects based on adaptive K-means algorithm using long-term ACF data^1^, (2) IHS identification based on multi-features and empirical threshold algorithm, (3) IHS identification based on nighttime light data, (4) Validation of the final GIHS data.

#### Construction of heat source objects

The remote heat source object detection model using real-time VIIRS active fire/hotspot data static and persistent IHS in the ACF time series were found to be tightly distributed around their hot centers due to position invariance and temporal consistency. The K-means algorithm, as a simple cluster algorithm, was used to cluster fire hotspots based on their spatial location. Then, a heat source object detection model based on adaptive K-means algorithm^1^ was adopted using long-term ACF data ranging from 19 January 2012 to 31 December 2021 (3633 days). In this part, a total of 35,474,293 fire hotspots were segmented into 11,271,322 heat source objects.

#### IHS identification

IHS objects are static and persistent, while biomass fires are usually sparsely distributed. Then, multi-features including the ACF number, hotspot density per square kilometer, the time span information on heat source objects was extracted. Empirical threshold algorithm^1^ using multi-features were used to distinguish IHS objects from all heat source objects and remove biomass fires objects.As a result, 29,534 IHS were identified from 11,271,322 heat source objects.

#### IHS identification based on nighttime light data

IHS can be distinguished from most biomass fires. However, some fire-prone areas are inevitable. Some biomass rainforest fire areas near the equator have characteristics similar to those of IHS, with high incidence throughout the year and regional concentration. This phenomenon is more common in Brazil, Central Africa, and the South Sea Islands^32^. As such, the maximum nighttime light data between 2012 and 2021 were used to detect IHS in this paper^30^. Finally, 25,544 GIHS were identified from the initial 29,534 detected.

#### Validation of the final GIHS data

**Fig. 2:**
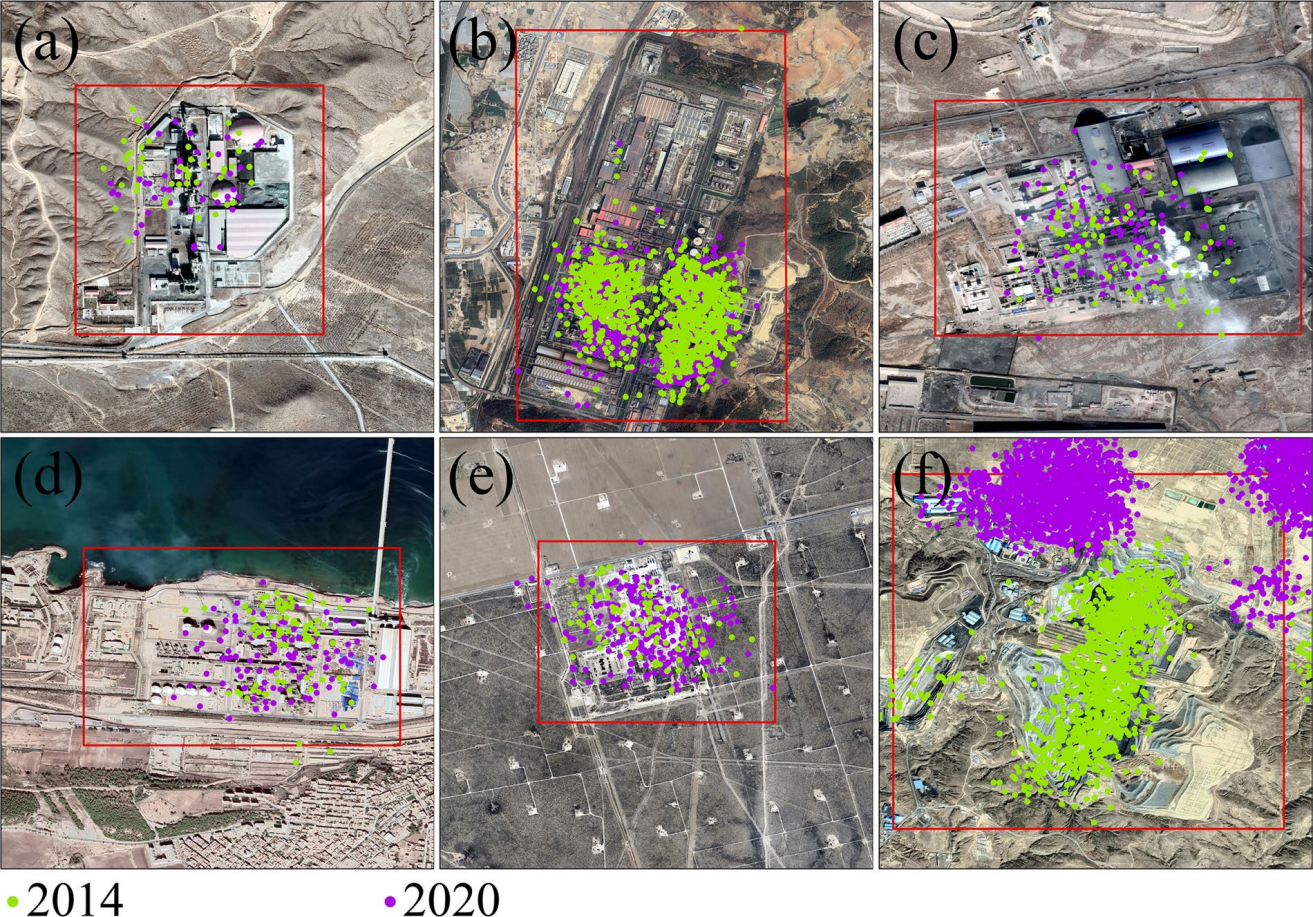
Characteristics of IHS in high-resolution remote sensing imagery (the green points represent the ACF in 2014, while the purple points represent the ACF in 2020). (**a**) Cement plants, (**b**) iron/steel facilities, (**c**) coking facilities, (**d**) oil refineries or chemical plants, (**e**) shale-gas development areas, and (**f**) coal mine development areas. The background imagery came from the Google Earth map (https://earth.google.com).

Considering that there is no complete and objective IHS especially with Geo-informarion in global, the User’s accuracy was used to to verify the long-term reliability of GIHS products for ACF^8,33^. All IHS objects were overlaid on online high-resolution image screenshots (e.g., Google Earth, AMAP, and Baidu Maps). Each IHS was manually checked individually based on online high-resolution image screenshots, POIs, and open street maps to distinguish the industrial backgrounds. IHS features stand out in high-resolution images. Cement facilities are frequently situated next to lime quarries and usually have tower-like preachers, long-stripe rotary kilns, dome-shaped raw-material reservoirs, and bowl-shaped clinker reservoirs (Fig. 2a). Iron/steel facilities often contain blast furnaces, gas tanks, electrical dust removal facilities, storage yards for coke, etc. (Fig. 2b). Storage yards with a dark backdrop are a common sight at coking facilities (Fig. 2c). Oil refineries or chemical plants frequently have an organized grid of storage tanks (Fig. 2d), and unconventional shale-gas development areas include well pads, ponds, and haulage roads for transporting water and chemicals (Fig. 2e). Traditional coal mine development areas are easily detectable as well (Fig. 2f). Craters, burned areas, and smoke plumes can also be used to distinguish between volcanoes and biomass burning regions.

Long-term ACF data on GIHS were simultaneously superimposed onto online high-resolution image screenshots to help verify IHS. Then, GIHS products were classified into 3 categories using the outcomes of manual verification. Real IHS that had been artificially confirmed was given the value 0, whereas non-IHS was given the value 1. 2 was an uncertain type since high-resolution images were unavailable at a certain period. In addition, Liu’s^8^ inventory was also verified in the above way. This reference data was used to validate our GIHS.

### Statistical analysis

The number of working IHS (NIHS) was extracted in the study area for various years. Additionally, the total number of ACFs in the working IHS (NFIHS) was also derived to represent the production scale of the IHS in the study area for different years. To extract the NIHS and NFIHS, we used statistical analysis to determine the trend of change and excluded the impact of anomalous fluctuations from the acquired GIHS time series data. The annual NIHS and NFIHS on the scales of latitudinal/longitudinal zones, continents and countries are summarized.

Although the interannual consistency has been maximized in the mapping framework described above, resource utilization and policy adjustment-related interannual fluctuations may still have an impact. The Theil-Sen estimator, Mann–Kendall test in area^34^ and linear slope^35^ were employed to estimate the long-term trend of change.

## Data Records

Our GIHS data records provide geometric location and attribute information for 25,544 IHS objects. All data records are freely available to the public in shape-file format at 10.5281/zenodo.8308133^36^. Its coordinate system is the World Geodetic System 1984 (WGS84) datum.

GIHS dataset contains 19 main attributes, including ‘FID’, ‘Locations’, ‘Points_num’, ‘Min_date’, ‘Max_date’, ‘Type’, ‘CONTINENT’, ‘Nation_Name’, ‘date20××_p’, and ‘area’ (Table 1). ‘date20××_p’ is the number of active fire/hotspot data within the industrial heat source in year ‘20××’ (belonging to a set of values [2012, 2013, 2014, 2015, 2016, 2017, 2018, 2019, 2020, 2021]). The fields of ‘Min_date’ and ‘Max_date’ can be used to describe the operating status and duration of the industrial heat source. The following criterion, “date20××_p” > 0, can be used to find IHS items that will function in a year. Additionally, the value of “date20××_p” can show the IHS’s operational state or heat radiation fluxes. If “date20××_p” is zero, this IHS did not operate during the “20××“ year. If the “date20××_p” value is higher than the previous year, the IHS’s production or operational efficiency was higher. Otherwise, efficiency was worse.

**Table 1:** Attribute descriptions of GIHS datasets.

No.	Name	Type	Note
1	FID	int	Unique identification number of an IHS
2	Locations	Polygon	An area polygon to represent the boundaries and geometric locations of an IHS
3	Points_num	int	Number of active fire/hotspot data in an IHS during 20 January 2012 to 31 December 2021
4	Min_date	String	The earliest date of active fire/hotspot data in an IHS during 20 January 2012 to 31 December 2021. The date format is ‘YYYYMMDD’.
5	Max_date	String	The latest date of active fire/hotspot data in an IHS during 20 January 2012 to 31 December 2021. The date format is ‘YYYYMMDD’.
6	CONTINENT	String	The continental Name in which an IHS is located. It belongs to a set of values {‘Asia’, ‘Africa’, ‘North America’, ‘South America’, ‘Europe’, ‘Oceania’, ‘Sea’}, where ‘Sea’ refers to an IHS located on the ocean.
7	Nation	String	The national Name in which an IHS is located.
8	Type	String	The type identification number of an IHS, belonging to [0, 1, 2], where 0 represents an IHS verified as an IHS, 1 as not belonging to an IHS, and 2 uncertain IHS type.
9	date20××_p	int	Number of active fire/hotspot data within an IHS in a year ‘20××’. With ‘20××’ belonging to a set of values [2012, 2013, 2014, 2015, 2016, 2017, 2018, 2019, 2020, 2021]
10	Area	float	The area of an IHS, its unit is square kilometers (km^2^)

## Technical Validation

### Detection efficiency of GIHS

A total of 25,544 IHS objects were obtained in our GIHS dataset. Among all IHS objects, 23,232 (90.95%) objects were verified by humans based online Google Earth data or high-resolution remote sensing images, 1671 (6.54%) were non-his objects, and 642 (2.51%) remained undetermined since high-spatial resolution remote sensing data were unavailable. The total user’s accuracy of our GIHS inventory is between 90.95% (23231/25544) and 93.46% (23873/25544). The Asian continent had the greatest number of IHS, with 10,465 representing 45.05%, followed by Europe and North America. 78% (19974/25544) of GIHS is located in Asia, Europe, and North America as shown in Table 2.

**Table 2:** Statistical results of GIHS from long-term ACF data at the continental scale.

Continent	0	1	2	Total	Lower accuracy (%)	Upper accuracy (%)
Africa	1644	73	176	1893	86.85	96.14
Asia	9770	576	119	10465	93.36	94.50
Europe	4731	266	248	5245	90.20	94.93
North America	3943	259	62	4264	92.47	93.93
South America	1403	430	34	1867	75.15	76.97
Oceania	322	61	3	386	83.42	84.20
Other	1418	6	0	1424	99.58	99.58
Global	23232	1671	642	25544	90.95	93.46

Real IHS that had been artificially confirmed were given the value 0, whereas non-IHS was given the value 1. 2 was questionable since high-resolution photos were unavailable for a certain period. The ‘Other’ continent value means that those IHS were not located in any countries according to administrative boundaries. Most of the ‘Other’ IHS occurred on oceans or lakes.

**Fig. 3:**
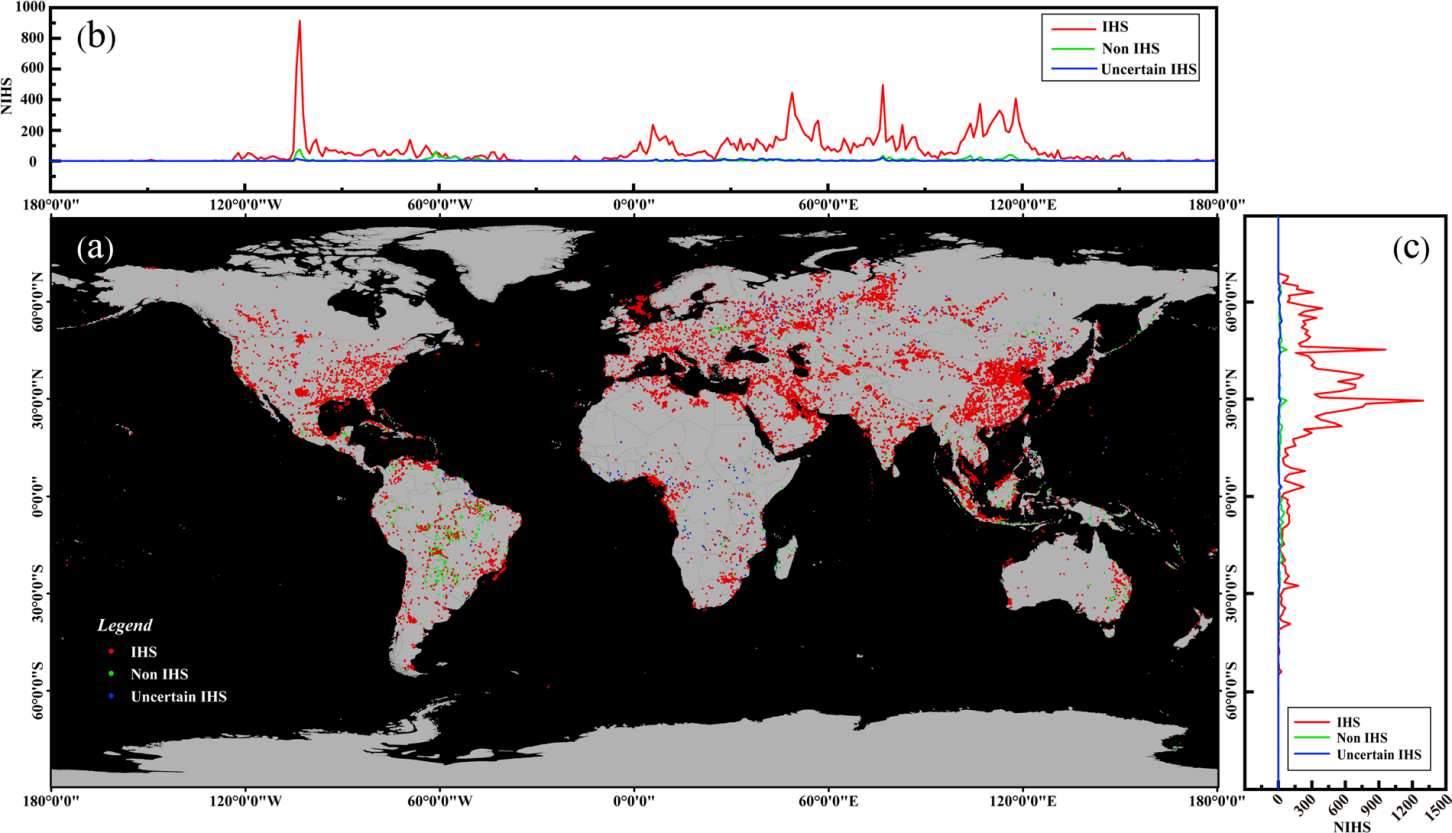
Distribution of 25,544 GIHS between 2012 and 2021 (legend annotated below the figures) derived from 3633 days of the ACF product. (**a**) Distribution of IHS by longitude. (**b**) Global spatial distribution of IHS. (**c**) Distribution of IHS by latitude.

The spatial distribution of 25,544 GIHS (Fig. 3) revealed that IHS was mainly focused in Asia, Europe, and North America, especially in Central-Eastern China, Khanty-Mansiysk and Yamal-Nenets, the Persian Gulf Area, Middle East America, Strait of Malacca, and North Bay. Most non-IHS were located in South America. According to latitude statistics, 89.95% of the IHS is located in the Northern Hemisphere, with approximately 56.16% distributed between 28°N and 48°N (primarily in China, the United States, etc., totaling 11,736), 19.94% distributed between 0°N and 28°N (totaling 4,633), and 48°N to 90°N accounting for 4,608. In terms of longitude, there are five main statistically significant peak ranges: 105°W to 95°W (United States region, totaling 1,352), 2°E to 12°E (Europe, Africa, totaling 2,409), 45°E to 55°E (Europe and West Asia, totaling 3,394), 75°E to 85°E (Russia, India, totaling 2,741), and 100°E to 120°E (China, Southeast Asia, totaling 7,895).

### Trends seen in the GIHS dataset

**Fig. 4:**
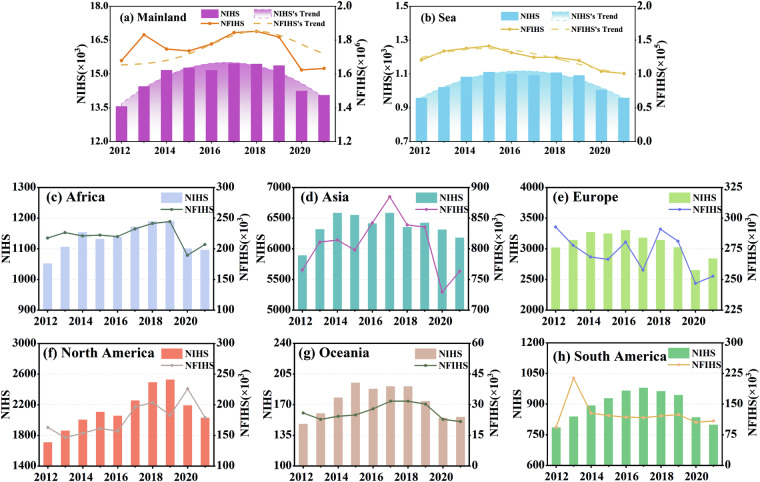
Temporal trend of extended time series (2012–2021) GHIS between NIHS and NFIHS. (left) The NIHS values present in different years. (right) The NFIHS values present in different years. (**a**) The trend of GHIS in the mainland, (**b**) The trend of GHIS in the sea, (**c**) Africa, (**d**) Asia, (**e**) Europe, (**f**) North America, (**g**) Oceania, (**h**) South America.

We analyzed global, continental and national trends of NIHS and NFIHS. The highest values of NIHS and NFIHS in the mainland was observed in 2017–2018, but it has been declining since 2018 in Fig. 4(a,b). The trends of these two variables are similar: they all increased from 2012, reached a minor peak in 2013, then declined, then reached their lowest point in 2015, and then began to increase again. NIHS on the sea exhibited a slow increase from 2012 to 2015, followed by a fluctuating downward trend after 2015. The maximum value for the NFIHS on the sea also occurred in 2015, but it has been decreasing since then.

To analyze the HIS variation trends across different continents, we divided the globe into six continents and seas and calculated the NIHS and NFIHS values for each continent from 2012 to 2021, as shown in Fig. 4(c–h). Based on the data, the following conclusions can be drawn: among these six continents, two general patterns of change can be observed. The first pattern is characterized by a rapid increase prior to 2014 or 2015 followed by fluctuating declines. This pattern occurs in Asia, Australia, Europe, and the offshore region. The second pattern exhibits a slow increase until approximately 2018, followed by a sharp decline. This pattern is seen in North America, South America, and Africa.

Furthermore, we employed Theil-Sen estimators and linear slope methods to calculate the trends of NIHS and NFIHS for each continent, and the reliability of the trends was assessed using the MK test (Table 3). NIHS trends in Africa, Asia, South America, Australia, and the sea are relatively stable, while the NFIHS shows a decreasing trend (excluding Africa and Australia). South America is the only continent where both NIHS and NFIHS exhibit increasing trends. Europe is the only continent where both NIHS and NFIHS show decreasing trends.

**Table 3:** Statistical results of change analysis for IHS (continental scale).

Continent	Slope		T_Slope		T_low__Slope		T_up__Slope		MK_Trend	
	NIHS	NFIHS	NIHS	NFIHS	NIHS	NFIHS	NIHS	NFIHS	NIHS	NFIHS
**Africa**	5	−101	7	1306	−89	−54914	54	17463	no trend	no trend
**Asia**	8	−1930	−22	−237	−230	−105980	428	45518	no trend	no trend
**Europe**	−41	−2813	−38	−3535	−372	−34470	188	33303	no trend	no trend
**North America**	56	6142	83	6753	−338	−47010	238	43374	increasing	increasing
**South America**	3	−3897	6	−2194	−110	−85594	54	119863	no trend	no trend
**Oceania**	0.05	99	0	2	−19	−7468	18	3774	no trend	no trend
**Other**	−0.51	−3259	−1	−3586	−86	−15987	64	13143	no trend	decreasing
**Global**	30	−6676	55	−2269	−1125	−194222	885	152736	no trend	no trend

**Annual Slope_NWH** and **Slope_NFHWH** changes are given by linear slope, slope_Theil_sen and its 95% confidence interval obtained by the Theil–Sen estimator, p value, and trend information from a Mann–Kendall test.

**Fig. 5:**
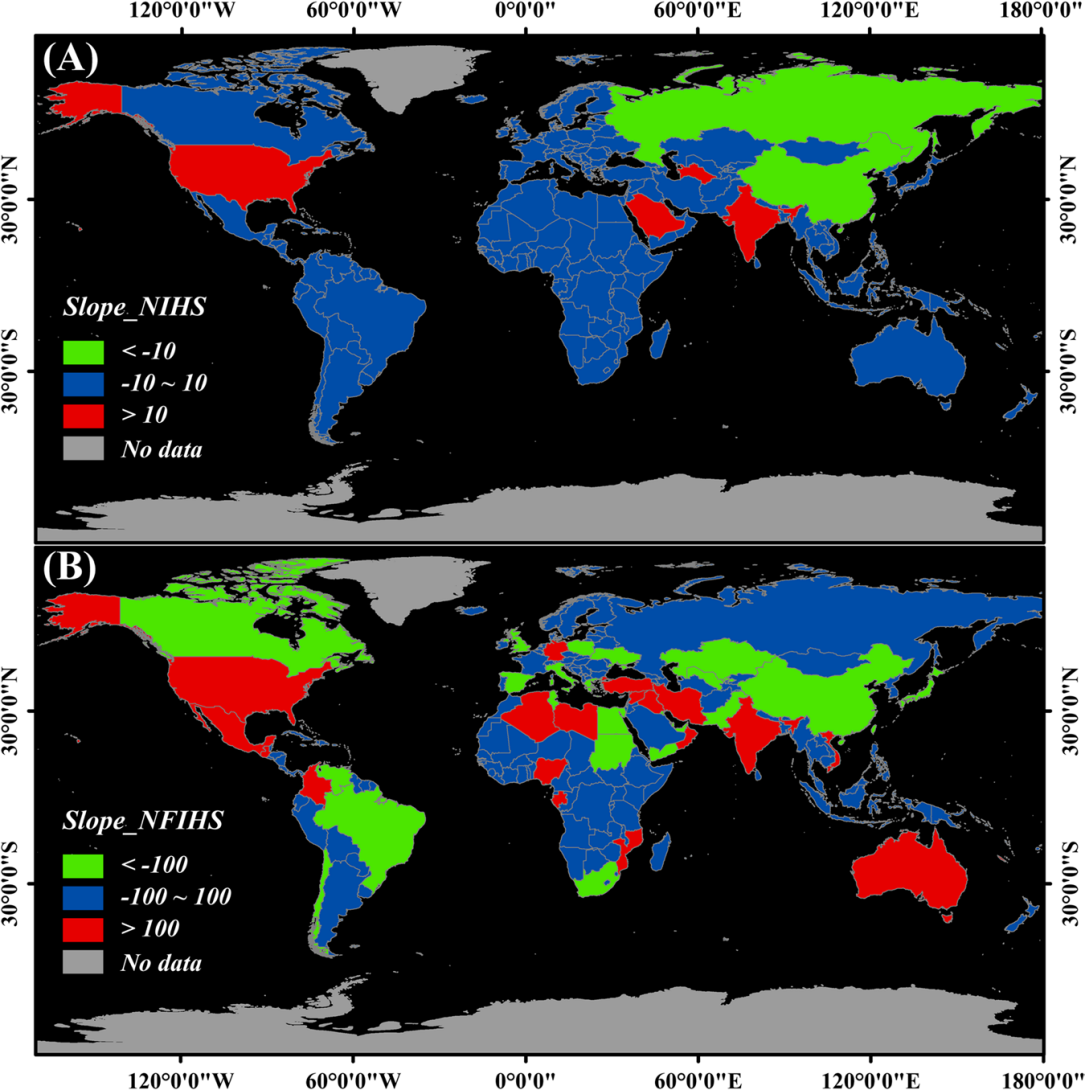
Changes in IHS at the national scale from 2012 to 2021. (**a**) The Slope_NIHS for different countries. (**b**) Slope_NFIHS for different countries.

On the national scale, the distribution of slopes of the NIHS and NFIHS were shown in Fig. 5. It was found that 85 countries exhibited an increasing trend in NIHS, 70 countries showed a decreasing trend, and 99 countries demonstrated a stable trend. Figure 5(a) illustrates countries with significant variation trends (with positive slopes greater than 10 and negative slopes smaller than −10). Among them, the United States, Saudi Arabia, India, and Turkmenistan exhibited an increasing trend in NIHS, indicating an increase in IHS in these four countries from 2012 to 2021. Conversely, China and Russia showed a decreasing trend in NIHS, indicating a decline in IHS during the same period. Regarding NFIHS (Fig. 5(b)), the United States remained the country with the fastest-growing trend, followed closely by Iran and Iraq. On the other hand, China, Venezuela, and Kazakhstan exhibited the fastest declining trends.

### Comparison with existing IHS datasets derived from the VIIRS Nightfire product

**Fig. 6:**
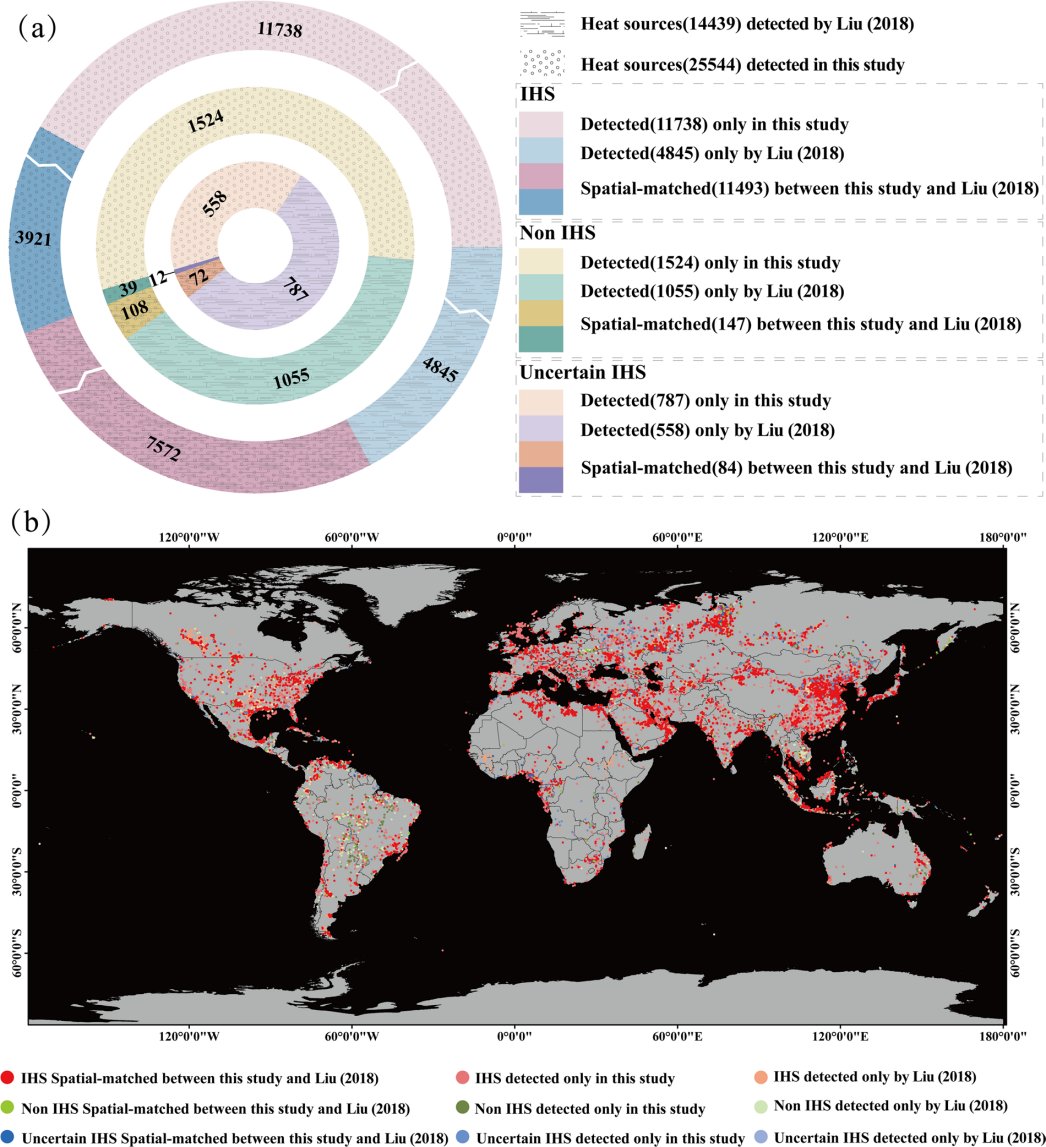
Comparison of our results with IHS detected by Liu *et al*.^8^. (**a**) Statistical comparison of the two datasets. (**b**) Comparison of the spatial distribution of matches and mismatches between the two datasets.

An object-oriented approach was introduced by some researchers for the segmentation and classification of various IHS using the VIIRS Nightfire (VNF) product (Liu *et al*.^8^; Sun *et al*., 2018). A total of 14,439 industrial heat sources operating between 2012 and 2017 were obtained from Liu’s product. Manual verification resulted in the inclusion of 12,417 industrial heat sources, 1,163 indeterminate sources and 859 nonindustrial heat sources (Fig. 6(a)). To verify the user’s accuracy and precision of our dataset, a comparative analysis was conducted between our GIHS data and Liu’s inventory in 2018 during the same period. The results of this comparison demonstrate that our dataset has significant improvements in the identification accuracy, number, granularity, and spatial overlap (Fig. 6(b)).

Firstly, Our dataset detected a substantially greater number with higher user’s accuracy of industrial heat sources, totaling 23,231 compared to the 12,417 accounted for in Liu’s study, thereby marking an increase of 87.10%. The identification user’s accuracy of our dataset was also higher, with an user’s accuracy rate of 90.95%~93.46% compared to Liu’s 86.00%~91.95%. The primary regions where both products misidentify IHS are largely situated within the agricultural straw burning areas of East Asia, North America, and South America. Secondly, the granularity of our dataset was higher, and the identification of IHS was more precise. The average area of each IHS identified by our dataset was 0.75 km^2^, while Liu’s average was 3.55 km^2^. Thirdly. the spatial overlap between our dataset and Liu’s was 60.98% (7572/12417), signifying an 11.51% increase compared to Liu’s coverage of our dataset (11493/23231). Lastly, our dataset with 19 characteristics has much more information than Liu’s inventory with 2 characteristics. In particular, the characteristics of “ACF_20××” can easily display the yearly operational status of the IHS. However, it is unavailable for Liu. Notably, there are still 4,845 IHS detected by Liu’s inventory and 11,738 IHS detected only by our GIHS.

### Comparison with existing single-IHS category datasets derived from Elvidge and GeoAsset

Gas flaring, iron and steel plants, and cement plants are all the main IHS objects. Elvidge’s inventoryand GeoAsset^33,37^ (combing iron/steel and cement production assets, McCarten *et al*.^38,39^ were compared with our GIHS dataset.

**Fig. 7:**
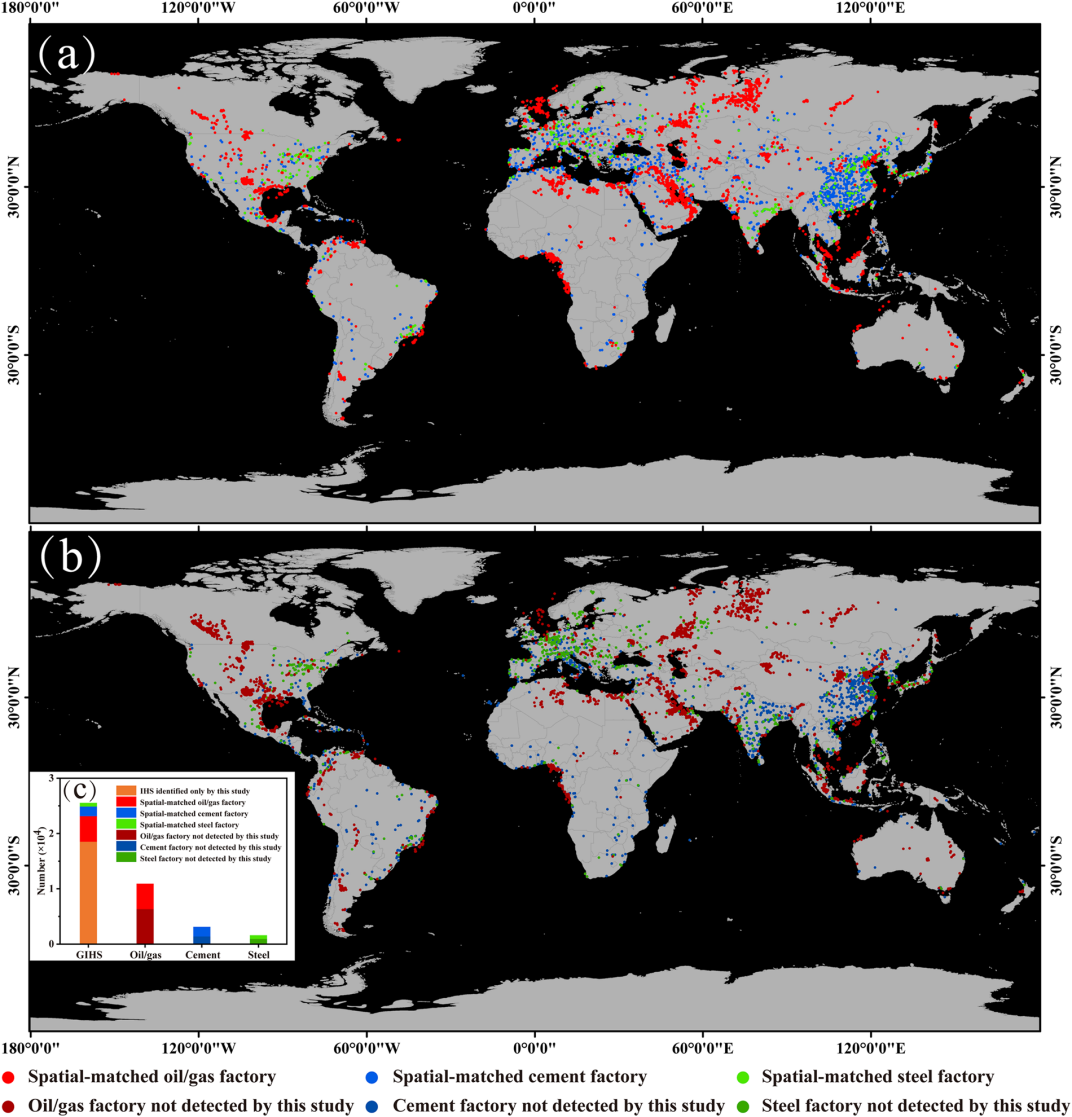
Comparison of our results with IHS detected by Elvidge (2015) and GeoAsset (2021). (**a**) IHS spatial matches to the GIHS, (**b**) IHS mismatches to the GIHS, (**c**) Statistical comparison of the four datasets.

The results show that the spatial matching rate for the oil/gas category of heat sources is 41.67% (4,628/11,108). Among the unmatched heat source objects, they are mainly distributed in shale gas development areas in North America, such as Alberta in Canada, North Dakota, Ohio, Pennsylvania, and Texas in the United States, as well as in the Persian Gulf region and Russia. For the cement production plant category, the spatial matching rate is 55.67% (1,735/3,117), predominantly located in East China and India. For the iron and steel production plant category, the spatial matching rate is 41.68% (666/1,598), primarily distributed in Western Europe and the Western United States (Fig. 7).

The unmatched IHS can be attributed to the following causes.The dataset provided by the GeoAsset project is based on machine learning and remote sensing image recognition techniques using Sentinel-2 MSI to locate cement and iron/steel production plants37, totally different mechanism and method with our and Liu’s methods. So, this method may result in mis-identification of non-plant areas due to similar image features. Additionally, some permanently closed or abandoned plants were included in the GEOAsset results since image-based recognition systems were unable to verify the operational condition of the plants.

## Usage Notes

When using GIHS dataset or part of it, please cited this manuscript. Te primary data format is shape-file, which contains a collection of files with 7 extensions (.shp, .shx, .dbf, .sbn, .sbx, .xml, .prj) that work together to store the geometric location and attribute information of GIHS polygon geographic features. Shape-files are widely supported and can be read or edited by various GIS software programs, such as Esri’s ArcGIS, QGIS, and GRASS GIS. Code Availability.

## Data Availability

Te create GIHS code notebooks are available from the GitHub https://github.com/ccmch/industry.
